# Corrective saccades influence velocity judgments and interception

**DOI:** 10.1038/s41598-019-41857-z

**Published:** 2019-04-01

**Authors:** Alexander Goettker, Eli Brenner, Karl R. Gegenfurtner, Cristina de la Malla

**Affiliations:** 1 Abteilung Allgemeine Psychologie, Justus-Liebig University Giessen, 35394 Giessen, Germany; 2 Department of Human Movement Sciences, Institute for Brain and Behavior Amsterdam, Vrije Universiteit Amsterdam, Amsterdam, The Netherlands; 3 Vision and Control of Action (VISCA) Group, Department of Cognition, Development and Psychology of Education, Institut de Neurociències, Universitat de Barcelona, Barcelona, Spain

## Abstract

In daily life we often interact with moving objects in tasks that involve analyzing visual motion, like catching a ball. To do so successfully we track objects with our gaze, using a combination of smooth pursuit and saccades. Previous work has shown that the occurrence and direction of corrective saccades leads to changes in the perceived velocity of moving objects. Here we investigate whether such changes lead to equivalent biases in interception. Participants had to track moving targets with their gaze, and in separate sessions either judge the targets’ velocities or intercept them by tapping on them. We separated trials in which target movements were tracked with pure pursuit from trials in which identical target movements were tracked with a combination of pursuit and corrective saccades. Our results show that interception errors are shifted in accordance with the observed influence of corrective saccades on velocity judgments. Furthermore, while the time at which corrective saccades occurred did not affect velocity judgments, it did influence their effect in the interception task. Corrective saccades around 100 ms before the tap had a stronger effect on the endpoint error than earlier saccades. This might explain why participants made earlier corrective saccades in the interception task.

## Introduction

The analysis of visual motion is crucial for our interactions with the environment, allowing us to deal with tasks such as catching a ball or judging the velocity of an approaching car. When objects are moving we almost automatically direct our gaze towards them^1^, especially if we want to interact with such objects^2,3^. Looking at moving objects has several advantages. First of all, tracking a moving object with the eyes maximizes the spatial resolution near the object^4^. Pursuing a moving object also improves our ability to predict how the object will continue moving^5^, as well as our ability to intercept the object at the correct position and time^3,6^. Moreover, tracking moving targets makes velocity judgments more invariant with respect to the sensory characteristics of the targets^7^ and can thereby reduce the effect of perceptual errors in interception^8^.

Moving targets are often not tracked completely smoothly, but with a combination of periods of smooth pursuit interspersed with corrective saccades that relocate the target onto the fovea^9^. Previous research on motion perception has mainly focused on how we can compensate for retinal slip caused by our own pursuit eye movements^10–12^ and on how motion perception and pursuit eye movements are related (for reviews see^13,14^). Little is known about the possible role of saccadic eye movements in judging how targets move (but see^15,16^). Goettker *et al*.^16^ have recently shown that the occurrence of saccades (and their size) is correlated with the perceived velocity of the object, while that is not the case for differences in eye velocity during pursuit. When tracking consisted of a combination of pursuit and an additional corrective forward saccade (a saccade in the direction of the target’s movement), the target was perceived to move faster than in the absence of such a saccade. In contrast, targets tracked with an additional corrective backward saccade (saccades in the opposite direction than the target’s movement) were perceived to move slower than targets tracked with smooth pursuit. If differences in perception were at the root of the differences in oculomotor behavior, a trial that is perceived to move faster would be tracked with a high pursuit gain and be most likely to be accompanied by backward saccades, which is the opposite of what Goettker *et al*.^16^ observed. Thus, the way we move our eyes to track a moving object seems to influence the object’s apparent velocity, rather than the other way around.

Misperception of target motion leads to errors in our actions directed at the targets^17^. Thus, if differences in the way we track moving targets lead to erroneous judgments about how fast the targets move, these differences in oculomotor behavior might also lead to errors in actions directed at such targets. The goal of this study is to investigate whether a change in the perceived velocity of a target as a result of a corrective saccade influences how people try to intercept the target. To test this, participants had to track moving dots with their eyes and either judge their velocity or intercept them by tapping on them. We manipulated the oculomotor behavior by using target movements for which the participants would spontaneously track differently on different trials (see^16^). We hypothesized that the different eye movement behavior on different trials would influence the targets’ apparent velocity and therefore lead to related systematic errors in interception. The results might provide interesting insights about how information is combined for different tasks, such as judging the velocity of a target and intercepting the same target with the hand. While information is probably integrated across the whole trial for perceptual judgments, the final endpoint error in interception behavior probably mainly depends on the perceived position and velocity roughly 100 ms before the tap^17–21^.

## Methods

### Participants

Two authors and nine naïve participants took part in the study (age range 27–60, 4 male, 1 left-handed). All participants had normal or corrected to normal vision. None had evident motor abnormalities. All participants gave written informed consent before taking part in the experiment. The experiment was approved by the ethical committee of the Faculty of Behavioural and Movement Sciences at the Vrije Universiteit Amsterdam. The experiments were carried out in accordance with the approved guidelines.

### Apparatus

**Figure 1:**
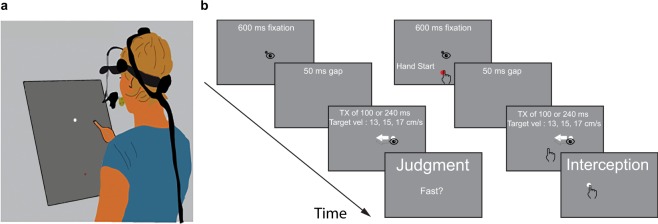
Method. (**a)** Depiction of the setup for the interception task. The white and red dots represent the target and the hand’s starting position, respectively. In the judgment task the setup was identical except that the participant’s hand was next to a keyboard rather than being equipped with a marker, and the starting position for the index finger was absent. (**b**) Paradigm of a single trial in the judgment (left) and interception (right) tasks. After an initial fixation of 600 ms the fixation point (black dot) disappeared and 50 ms later the target appeared with an offset either to the left or to the right. The magnitude of the offset (target crossing time, TX) was manipulated so that the target would pass the initial fixation location after 100 or 240 ms. Thus, the size of the step depended on the target’s velocity and the target crossing time. The target kept moving for 600, 650 or 700 ms. In the judgment task participants had to decide whether they had been presented with a fast or slow target on each trial. In the interception task they had to try to tap on the target with their right index finger before it disappeared.

The experiments were conducted in a normally illuminated room. During the experiment, participants stood in front of a large screen (Techplex 150, acrylic rear projection screen; width: 1.25 m; height: 1.00 m; tilted backwards by 30°) onto which stimuli were projected (In-Focus DepthQ Projector; resolution 800 by 600 pixels; screen refresh rate: 120 Hz). Participants had a clear view of the stimulus, as well as of their arm, hand and finger. They were not restrained in any way in either of the tasks (Fig. 1a).

We recorded both eyes’ movements with a head-mounted eye-tracking system (Eyelink II, SR Research) at 500 Hz. Participants held a biteboard with a dental imprint with their teeth. Three infrared markers were attached to the biteboard and monitored by an infrared camera (Optotrak 3020, Northern Digital) that was positioned at about shoulder height to the left of the screen. This allowed us to record the position of their head at 250 Hz. Where participants were looking on the screen was determined by combining the measurements of eye in head orientation from the eye tracking system with the position of the eyes and orientation of the head from the recorded marker positions (see *Calibration and synchronization*).

We used a computer keyboard to register the perceptual responses in the velocity judgment task. For the interception task, we attached an infrared marker to the nail of the index finger of the dominant hand. This marker was also tracked by the Optotrak camera at a frequency of 250 Hz.

### Calibration and synchronization

In order to relate the gaze and hand measurements to positions of stimuli on the screen, we needed to first know the spatial coordinates of the images on the screen. We used a pointer consisting of a rod with a tapered end and three infrared markers attached to the other end to calibrate the screen. Prior to calibration of the screen we calibrated the pointer by placing an additional marker at the tip of the tapered end so that we could determine its position with respect to the three other markers. The rendering of images on the screen was then calibrated by placing the tip of the pointer at five consecutively indicated image positions on the screen. The coordinates of the image positions were determined from positions of the three markers attached to the pointer.

The pointer and calibrated screen were used to determine the positions of the eyes relative to the biteboard. We placed the pointer between the screen and the participants, and we asked the participants to look with one eye and move however they wanted until the tip of the pointer was seen to be aligned with a small white dot presented on the calibrated screen. Once they considered the tip of the pointer to be aligned with the dot, they had to press the button of a mouse that they were holding in their hand. They were required to be static at the moment of recording their answer (move less than 1 mm during the last 300 ms). If they were static at the time, a new dot appeared at a different position. Otherwise, they had to press again to record their answer. Participants had to align the tip of the pointer with 20 dots using only the left eye and then with 20 dots using only the right eye. At the moment they pressed the mouse button the positions of the markers of both the biteboard and the pointer were recorded. We converted the coordinates of the tip of the pointer and of the dot on the screen into a line with respect to the markers attached to the biteboard. Thus, we got 20 different lines for each eye (one for each dot that the participants aligned). The position with respect to the biteboard that minimized the sum of the distances to all lines was considered to be the position of the eye. From that point on, we could determine the positions of the two eyes from measured positions of the markers on the biteboard.

Once we knew the positions of the eyes with respect to the biteboard, we calibrated the eye movements recordings. To do so, we presented a dot at the center of the screen, and participants had to move their heads for 30 s while maintaining fixation on the dot. By combining the coordinates of the pupil with respect to the head from the Eyelink data with the position of the dot relative to the eye in the head (based on the calibrated screen and the biteboard marker coordinates), we determined the scaling of Eyelink coordinates that minimized the deviations in calculated gaze position throughout this period for each eye. To verify the calibration, we rendered dots at the positions at which we considered the participants to be looking and asked the participants whether the dots were approximately at those positions. If so, the calibration was correct. If not, the calibration was repeated.

The final step in the calibration was to relate the position of the fingertip marker in the interception task to where the participant perceived his or her finger to be relative to the projected images on the screen. For this, we measured the position of the marker on the fingertip when the participants placed the fingertip at four indicated positions on the screen. With this calibration we corrected for the fact that the marker was attached to the nail rather than to the tip of the finger.

To synchronize the Optotrak recordings with the images appearing on the screen we flashed a disk in the upper left corner of the screen whenever a target appeared. A photodiode that was directed towards that part of the screen was used to briefly inactivate an additional Optotrak marker attached to the side of the screen (using custom built hardware with a delay of 1 ms). Detecting this inactivation provided information (to within the 4 ms sampling interval) about when the target appeared relative to the movement data, and allowed us to determine that the average latency with which we could adjust the images to events extracted from the online Optotrak data was 24 ms. All delays were accounted for, both in the analyses and in the feedback provided during the trials (except that the feedback was only provided after this delay). The calibration of the eye tracker and fingertip marker were run prior to starting each block of trials (details below). The calibration of the pointer and biteboard was done once, and that of the screen was done at the beginning of each day of measurement.

### Stimulus and procedure

To investigate the effect of corrective saccades on perception and action we used a modified version of the classical Rashbass paradigm^22^ to be able to manipulate the oculomotor behavior. We decided to measure the perceived velocity and interception behavior in two separate tasks to make sure that the perceptual judgments were not influenced by the errors in the interception task. Participants randomly started with either the judgment or the interception task (Fig. 1b).

### Judgment Task

**Figure 2:**
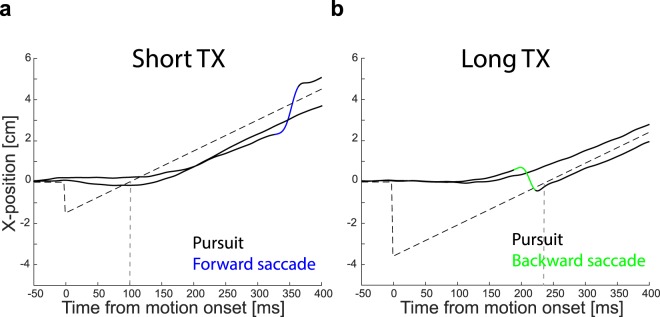
Examples of oculomotor behavior. **(a)** Gaze (thick continuous lines) across time for two representative trials for the short target crossing time condition for a target that moved at 15 cm/s. The target’s position is indicated by the dashed black line. The black color in the gaze indicates the initial fixation followed by smooth pursuit. The blue color indicates a forward saccade. Thus, one trial has a pure pursuit response, while the other contains a forward saccade. The dashed grey vertical line represents the target’s crossing time. (**b**) Representative trials for the long target crossing time. Again, one trial has only a pure pursuit response, whereas the other now has an additional corrective backward saccade, indicated in green. Other details as in a.

Participants were standing in front of the screen and had to fixate a 1 cm diameter black disk presented 5 cm above the center of the screen for 600 ms. The fixation disk disappeared for 50 ms and a 2 cm diameter white target disk appeared either to the left or the right of the fixation disk. The target disk moved for either 600, 650 or 700 ms at one of three different velocities (13, 15 or 17 cm/s) in the opposite direction than the target’s step (i.e. if the target appeared to the left of the fixation disk it moved to the right and vice versa). Participants were asked to follow the target with their eyes as accurately and smoothly as possible. The crucial variable that we manipulated was the time it took for the target to cross the initial fixation position, the so-called target crossing time (TX)^23,24^. To do so, we adjusted the size of the initial step based on the upcoming target velocity so that it crossed the initial fixation either after 100 ms (short TX) or after 240 ms (long TX). The short target crossing time was chosen so that the oculomotor behavior across trials was mainly a mix between pure pursuit and pursuit with additional corrective forward saccades. The long target crossing time was chosen to obtain a mixture between pure pursuit trials and trials with pursuit and additional backward saccades (Fig. 2). Once the target disappeared, participants had to judge whether the target was a fast or a slow one in comparison to all the other targets they had seen until then^25^ by pressing ‘1’ or ‘2’ on a computer keyboard that was placed to the right of their body. Each participant performed two blocks of 252 randomly interleaved trials (2 TX × 3 durations × 3 velocities × 2 directions × 7 repetitions of each combination). Participants could have a break anytime they wanted by delaying pressing the key to give their answer. Once they pressed the computer keyboard the next trial started. Each block lasted approximately 15 minutes.

### Interception task

The stimulus we used was the same as the one in the judgment task. However, in the interception task participants had to move the index finger of their dominant hand to a starting position to start each trial. The starting position was a 1.5 cm diameter red disk that appeared 20 cm below the screen center. Participants again were instructed to follow a target with their eyes, but now they had to try to intercept the target by tapping on it. Taps were detected online. A tap was considered to have occurred if the deceleration of the movement of the hand orthogonal to the screen was at least 50 cm/s^2^ while the finger was less than 5 mm above the screen. To avoid interpreting motion onset as a tap, we also checked that the finger was moving towards the screen, and that it had been lifted to at least 1 cm off the screen since being placed at the starting position. Feedback was provided after each tap. If participants hit the target, there was a sound and the target stopped moving. The target remained at the position at which it had been hit for 500 ms. If participants missed the target, it was deflected away from the finger at 100 cm/s, remaining visible for 500 ms. If no tap was detected online we looked at the raw Optotrak data offline. If we could find a point with a movement deceleration of at least 25 cm/s² in the direction orthogonal to the screen while the finger was less than 5 mm above the screen, this point was used as the tap. The number of trials per block was again 252. Participants could have a break anytime they wanted by not placing the index finger at the starting position. Every participant completed two blocks, each of them lasting for about 13 minutes.

### Data analysis

The data from both eyes were averaged to estimate the gaze position during the trial. Participants were standing in front of the screen, at whatever distance they felt would allow them to move freely and tap the screen comfortably. Given the different heights of the participants and the variations in distance across trials as a result of participants not being restricted in any way, we report the gaze velocity in cm/s on the screen rather than in deg/s. This allows us to meaningfully average the gaze data across trials and participants. In general, participants’ eyes were a bit more than 57 cm from the screen, so 1 cm on the screen is a bit less than 1 deg of visual angle. We performed an offline drift correction by averaging the mean gaze position for the 100 ms before the fixation target disappeared and subtracting the difference vector between this value and the position of the target at that time from the eye position vector throughout the trial. The median correction in the horizontal direction was a shift of 0.4 cm for the judgment task and 0.5 cm for the interception task. We then used a low-pass Butterworth filter with a cutoff frequency of 30 Hz to filter the horizontal gaze position during each trial. We computed the velocity by digitally differentiating the filtered eye position signals. The acceleration at each point during the trial was computed by dividing the difference between the velocities 10 ms before and 10 ms after the respective time point by the 20 ms between them.

Saccades were detected when the velocity of the eye was at least 2 times higher or 2 times lower than the target velocity (in the same or in the opposite direction than the target motion respectively) and had an acceleration of more than 300 cm/s². The onset of the saccade was defined as the moment at which the acceleration was first above the threshold. The offset of the saccade was defined as the moment after the peak velocity at which the slope of the acceleration switched its sign. This slightly underestimates the saccade duration, but it works reasonably well even when gaze velocity is adjusted after the saccade. Trials were inspected visually to make sure saccades were detected correctly. Pursuit onset was determined as the first time the eye velocity was higher than two times the standard deviation of the eye velocity during fixation (−50:50 ms after target movement onset) and stayed above 30% of the target velocity for 5 consecutive frames (10 ms). After the detection of saccades and pursuit onset we used an additional low-pass Butterworth filter with a cutoff frequency of 20 Hz to filter the velocity and linearly interpolated the velocity during saccades for some parts of our analysis.

For each saccade we extracted the latency as well as the amplitude. Saccades were classified as forward saccades if the direction of the saccade was the same as the target’s movement. If the saccade was in the opposite direction than the target’s movement, the saccade was classified as a backward saccade. We additionally measured the instantaneous eye crossing time (EX) for each saccade based on the paper by de Brouwer *et al*.^24^. The EX is defined as the gaze position error (gaze position-target position) divided by the retinal slip (gaze velocity-target velocity) measured 100 ms before the saccade onset. It is thought to be a correlate of a potential saccade trigger mechanism (see^26^).

For each trial in the interception task we determined the tapping error as the horizontal distance between the position at which participants tapped the screen and the position of the center of the target at the moment of the tap. We considered tapping ahead of the target to be a positive error and hitting behind the target a negative error, irrespective of the direction in which the target was moving. We concentrated on the horizontal error because the targets moved horizontally so any interception error caused by misperceiving the target’s velocity should be reflected in a horizontal error. Indeed, participants tapped close to the target center in the vertical direction (average across all trials 0.01 cm below the target center, standard deviation 0.42 cm) in all conditions. To observe whether the presence of saccades led to different kinematics during the interception movement we also calculated the hand velocity 100 ms before the tap from the recorded Optotrak data, which we had previously filtered in a similar manner as the eye movement data. As for the gaze, we used the mean of the velocity 120 to 80 ms before the tap as the estimate of the velocity 100 ms before the tap.

Each measurement was computed for each trial, and we divided trials into trials with pure pursuit and trials containing corrective saccades. To observe the effect of corrective saccades we computed the difference between performance on trials tracked with corrective saccades and performance on the same kind of trials when tracked with pure pursuit. We did this for each velocity and then averaged across the velocities for each participant. We compared the effect of forward and backward saccades with paired t-tests. Our hypotheses predict specific effects of the corrective saccades. We expect to see targets with forward saccades to be judged as being faster than targets with backward saccades. As a consequence of being judged as moving faster, we expect participants to hit ahead of the target when corrective saccades are forwards and behind the target when saccades are backwards. Finally, we predict an effect of the saccades on hand velocity, with higher hand velocities when the target is perceived to move faster. Since we have specific hypotheses about the direction of the effects we used one-sided paired t-tests to test the effects of corrective saccades. For the analysis of other potential differences in behavior (interception latencies, saccadic latencies and the EX of saccades) we used two-sided paired t-tests. To compare the influence of corrective saccades on the judgment and interception tasks we computed the correlation across participants between the effect of corrective saccades on the velocity judgment and on the endpoint error in the interception task.

### Exclusion of trials

For the eye movement analysis, we excluded trials in which we could not detect a pursuit onset or in which the latency of pursuit onset was shorter than 50 ms. Pursuit onsets shorter than 50 ms could indicate anticipatory pursuit. The randomization of the direction in which the targets moved prevented this from occurring frequently (it only happened in 56 and 81 trials in the Judgment and Interception tasks, respectively). We also excluded trials in which we detected blinks during the time the target was presented or when the position error (error between gaze and target) after the saccade was larger than 5 cm. Large position errors typically indicated that participants in this trial stopped tracking the target and already went back to the starting location. For the analysis of interception we excluded trials in which we could not detect a tap (502 taps were missed during the recording, but 141 of them could be identified offline and are included in the analysis) or in the rare case (35 trials) that the tap was more than 3 cm from the target (tapping too far from the target usually indicates that there was an error in detecting the tap).

We had to exclude the data of the judgment task of one participant because her tracking was incomplete. She always made a saccade back to the center before the target stopped moving. In the interception task she kept tracking the target until the end, showing again that people naturally look at moving objects during interception. Across all observers, we could use 4729 of 5040 trials (94%) in the judgment task and 4951 of 5554 trials (89%) in the interception task for the analysis.

## Results

We wanted to investigate whether changes in perceived velocity related to additional corrective saccades would lead to equivalent systematic errors in interception, quantified as the horizontal distance between the hit and the target position. To quantify the influence of corrective saccades we split the trials for each participant based on their oculomotor behavior (Fig. 2). We evaluated the effect of forward and backward corrective saccades by comparing trials with identical targets tracked with and without corrective saccades. We refer to the latter as having pure pursuit. We first looked at the results of the judgment task, to replicate the effect of corrective saccades on perceived velocity^16^. Then, we looked at the results of the interception task. In the end we related the findings of both tasks to investigate whether, and if so how, the perceptual effect translates into errors in the interception task.

### Judgment task

**Figure 3:**
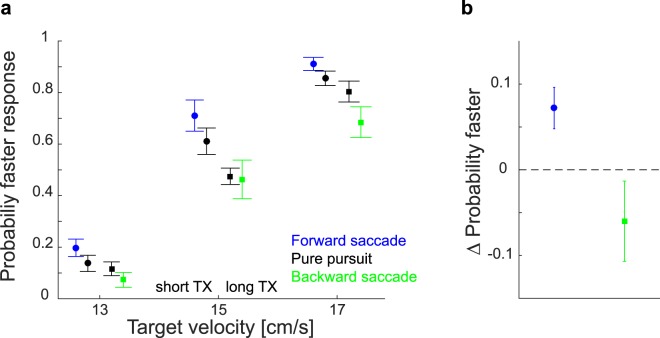
Effect of corrective saccades on velocity judgments. **(a)** Average of the participants’ mean velocity judgments for the three different target velocities and two values of TX, separated by the oculomotor behavior. Black symbols show responses on pure pursuit trials. Blue and green symbols show responses on trials with forward and backward saccades, respectively. Circles show the data for the short TX. Squares show the data for the long TX. The data points for each velocity have been spread horizontally to improve visibility. (**b**) Summary of the effect of corrective saccades on perceived velocity: difference between trials with corrective saccades and the corresponding pure pursuit trials (averaged across the three target velocities). All error bars show the standard error of the mean across participants.

Figure 3a shows the average probability of faster responses for the different target velocities, target crossing times and oculomotor responses. Within each target crossing time (TX) there is a systematic effect of the occurrence and direction of the corrective saccade. Trials tracked with additional forward saccades are perceived to move faster than the corresponding trials tracked with pure pursuit. In contrast, targets in trials with backward saccades are perceived to move slower than the corresponding targets in trials with only pure pursuit. We quantified the effect of making saccades by taking the difference between the average judgments of trials with corrective saccades and trials with pure pursuit for both TX, and averaging the values across the three velocities (Fig. 3b). In this way we could isolate the influence of corrective saccades as the presence of saccades was the only difference between the trials. Forward saccades led to a significantly faster percept than backward saccades (t_9_ = 2.612, p = 0.014), confirming that the occurrence and direction of corrective saccades lead to systematic changes in perceived velocity.

If one looks at the judgments of the pure pursuit trials for each velocity (black symbols in Fig. 3a), it can be seen that targets in short TX trials are also perceived to move slightly faster than those in long TX trials, although both are tracked with pure pursuit. This suggests that the participants’ judgments were influenced by the TX itself, in addition to the effect of the oculomotor behavior. Since the presentation durations were the same for both TX, this effect might be caused by the final position of the target. Targets in trials with short TX times travelled to more eccentric positions, which the participants might use as an additional cue for judging the velocity. Nevertheless, results show the predicted effect of the presence of corrective saccades on participants’ judgments of target’s velocity for each velocity and TX (for more details and control experiments see^16^).

### Interception task

For the interception task we performed similar comparisons as for the judgment task. We averaged performance for the different oculomotor responses and values of TX. To further investigate the temporal aspects of a possible role of corrective saccades on interception we determined the time it took participants to hit the target as well as saccadic latencies. We also measured the pursuit latencies and the eye crossing times (EX, see methods) for each kind of saccade to determine to what extent they can indicate when the decision was made to trigger saccades^24,26^. As a direct indicator of the velocity estimate used for the interception task, we also measured the hand velocity shortly before the tap, as the hand velocity seems to be related to the perceived target velocity^27^.

#### Interception Errors

**Figure 4:**
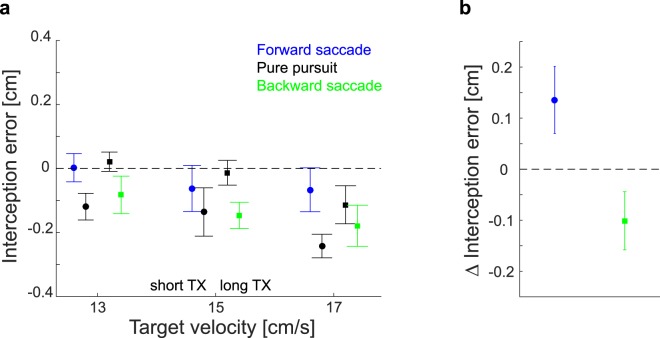
Effect of corrective saccades on interception. **(a)** Average lateral interception errors across participants for the three different target velocities and two target crossing times (TX) separated by the oculomotor behavior. Black symbols show responses for pure pursuit trials. Blue and green symbols indicate trials with forward and backward saccades, respectively. Circles show the data for the short TX. Squares show the data for the long TX. The data points for each velocity have been spread horizontally to improve visibility. An error of 0, shown by the black dotted line, would indicate tapping at the center of the target. Positive errors indicate hitting in front of the target center. (**b**) Summary of the effect of corrective saccades on interception errors: difference between trials with corrective saccades and the corresponding pursuit trials (averaged across the three target velocities). All error bars represent the standard error of the mean across participants’ average values.

In line with our perceptual findings, we observed that the errors participants made when hitting the targets were different in trials with additional corrective saccades than in trials with pure pursuit eye movements (Fig. 4a and b). Participants hit further ahead of the target in trials with forward saccades than in comparable trials in which the target was tracked with pure pursuit. In trials with backward saccades the taps were further behind the target than in trials with pure pursuit. This is what one would expect if targets are perceived to move faster and slower than they really are, respectively. We again quantified the influence of the saccades by taking the difference between trials with corrective saccades and pure pursuit trials. We found the same dependency on the direction of the saccade as we had seen for the judgment task (t_10_ = 2.138, p = 0.029).

As for the judgment task, there was a systematic effect of corrective saccades on the interception errors when comparing the corresponding trials. In addition to this effect there was again a difference between the errors when comparing the trials with pure pursuit and different target crossing times (Fig. 4a). This effect may be related to the differences in where participants intercepted the targets on the screen. Due to our stimulus design, long TX trials, which started further in the periphery, were intercepted closer to the center of the screen (average distance of 3.64 cm from the screen center) than short TX trials (average distance of 5.56 cm).

#### Interception Timing

**Figure 5:**
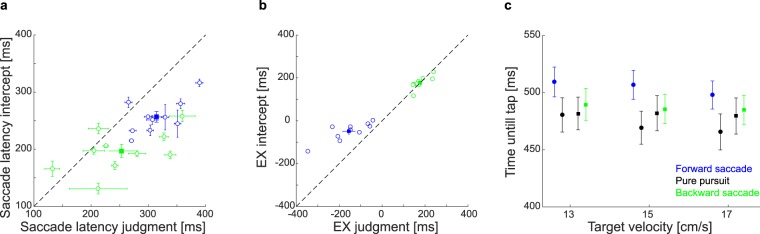
Times until saccade and tap. **(a)** Average saccade latencies in the judgment and interception tasks for forward (blue) and backward (green) saccades. Open circles show individual participants’ mean values and the respective standard error for each participant. The filled squares show the average saccade latencies and the standard error of the mean across participants. The black dashed line is the unity line. (**b**) Average eye crossing time (EX) for forward (blue) and backward (green) saccades for the judgment and interception task. Symbols and line as in panel a. Negative crossing times indicate that the target was ahead of the eye. Positive values indicate that the target was behind the eye. (**c**) Average time taken to hit the target for each target velocity, value of TX and kind of oculomotor behavior.

As interception is about being at the right place at the right time, we also looked at the timing of the oculomotor behavior and of the interception behavior. Having the same targets in the judgment and interception task allowed us to examine whether the timing of the corrective saccades was adjusted by the upcoming action. On average, saccades occurred significantly earlier in the interception task than in the judgment task (Fig. 5a). In the judgment task forward saccades occurred after an average latency of 314 ms and backward saccades after 253 ms. In the interception task, both types of saccades occurred significantly earlier: forward saccades occurred after 257 ms and backward saccades after 197 ms (t_9_ = 5.614, p < 0.001; t_9_ = 2.952, p = 0.016, respectively). To examine whether there was a selective change in the timing of the saccades or a general change in the oculomotor behavior we compared the oculomotor behavior of the two tasks in more detail. We determined the pursuit latency as well as the gaze position error 100 ms before the tap for each target crossing time. For the judgment task we determined the latter value at the average time of that participant’s taps in the interception task. We found that pursuit latencies, like the saccadic latencies, were shorter in the interception task (t_9_ = 4.885, p < 0.001 for trials with short TX and t_9_ = 7.066, p < 0.001 for trials with long TX), suggesting a more general reduction of the latencies of the oculomotor system. The position errors of the eye 100 ms before the tap were comparable for the two tasks (both p’s > 0.40), so the shorter latencies did not result in more accurate pursuit later in the movement.

To test whether the circumstances under which corrective saccades were initiated differed between the tasks, we computed the instantaneous eye crossing time (EX) before each saccade. This measure is similar to the target crossing time, but is calculated on the basis of the gaze position error and the retinal slip 100 ms before saccade execution (Fig. 5b). The EX is thought to be correlated with the decision of when to trigger catch-up saccades during pursuit^24,26^. The comparison revealed no significant differences between the tasks for backward saccades (t_9_ = 0.178, p = 0.862), but there was a significantly shorter EX in the interception task for forward saccades (t_9_ = 5.320, p < 0.001), suggesting that in the interception task forward corrective saccades were not only triggered earlier than in the judgment task, but also at moments at which the urgency of making a saccade is less evident. Maybe saccades occur earlier during interception trials to avoid having saccades close to the tap, which is an important moment for obtaining feedback^28^.

The time it took for participants to hit the target was longer for trials with additional corrective saccades than for trials with pure pursuit. This effect was especially pronounced for trials with forward saccades (Fig. 5c). On average, the targets in trials with forward saccades were intercepted after 503 ms (SEM = 12 ms), significantly later (t_10_ = 5.908, p < 0.001) than in trials with identical targets but pure pursuit (M = 472 ms; SEM = 14 ms). Trials with additional backward saccades were intercepted only slightly later than those with pure pursuit (487 ms vs 481 ms), and this difference was not significant (t_10_ = −1.266, p = 0.234). The shorter saccade latencies together with hitting the target later after having made a corrective saccade in the interception task, suggest that participants tried to maximize the time without a saccade before the tap. This effect was especially strong for forward corrective saccades, because forward saccades typically happen later and thus closer to the moment of the tap.

#### Hand Velocity

In line with faster targets being hit faster, the velocity of the hand at the crucial moment 100 ms before the interception was slightly faster for trials with forward saccades in comparison to the pure pursuit trials, whereas for backward saccades the opposite pattern was present. Forward saccades led to an average increase in hand velocity of 1.23 cm/s, whereas backward saccades led to an average decrease of hand velocity of −2.21 cm/s (the difference between the two was significant; t_10_ = 2.00, p = 0.037). Thus, changes in perceived velocity due to corrective saccades may not only influence the tapping error but also the kinematics of the interceptive movement.

### Relation between velocity judgments and interception

**Figure 6:**
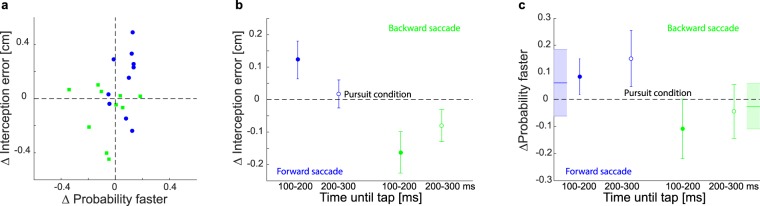
Relation between judgment and interception. Blue symbols show effects of forward saccades. Green symbols show effects of backward saccades. (**a**) Effects of corrective saccades on individual velocity judgments and interception errors. (**b**) Effects of corrective saccades on interception errors when only considering trials in which the corrective saccades occurred within the indicated time windows. The filled dots show the interception error for trials in which saccades occurred between 100 and 200 ms before the tap. The empty dots show the error when saccades occurred between 200 and 300 ms before the tap. Both are compared with errors when there were no saccades (pursuit condition; dashed line). Error bars show the 95% confidence interval estimated using bootstrapping. (**c**) Effects of corrective saccades on ‘faster’ judgments for equivalent time windows to those used for the interception task. Note here that as there was no tap in the judgment task, we used the average time until tap for each participant in the interception task to align the time windows in a similar manner. The shaded lines at the edges of the figure show the effects of corrective saccades happening during the last 100 ms before the end of the presentation. Probabilities are expressed as differences from those in the pure pursuit condition. Values are averages across the three target velocities. The error bars represent 95% confidence intervals based on the variability across participants.

We found similar saccade-related biases in visual judgments and in interception, but there was no evident correlation between the two biases across participants (r_10_ = 0.25, p = 0.47 for forward saccades; r_10_ = −0.02, p = 0.94 for backward saccades; Fig. 6a). The lack of correlation could just reflect the variability in the data, but it could also be due to differences between the use of information for the two tasks. For the interception task, participants may rely more on information at a certain moment. For instance, they can only use information until about 100 ms before the moment of the tap, because from that moment they can no longer make online corrections to the ongoing movement^18^. In contrast, information can be integrated across the whole trial to judge the velocity. We therefore examined whether the time of the corrective saccades influences the interception or judgment. We compared two time windows, one that is likely to be crucial for interception (saccades 100 to 200 ms before the tap, at the last moments at which information can be used to guide the action), and one that is probably less important (saccades 200 to 300 ms before the tap, leaving time to respond to information after the saccade). We selected all trials within those time periods, irrespective of the participant, because otherwise we would not have enough trials since participants seem to suppress saccades shortly before the tap. We found that, when saccades occurred closer to the tap, they had a stronger effect on the interception error (Fig. 6b). In the judgment task we performed a comparable analysis. For each participant we selected trials with corrective saccades in the same time windows as in the interception task. To compare the influence of corrective saccades at similar times we took the average time until tap for forward and backward saccades in the interception task and aligned the time windows on these values for the judgment task. We computed the average velocity judgment for each target velocity for the two different time windows and compared it to the average judgment for pure pursuit trials. For the judgment task we observed no systematic modulation depending on the timing of the corrective saccade (Fig. 6c). One could argue that the time windows are not directly comparable between the tasks, as in the judgment task the information of the whole trial could be used for the velocity judgment. To test that the stronger influence of late information is not purely a recency effect, we also calculated the effect of corrective saccades happening during the last 100 ms before the trial end in the judgment task, but did not observe a systematically larger difference than in the other time windows for this time period (Fig. 6c).

Taken together our results suggest that corrective saccades influence the perceived velocity of the target. The interception error is modulated by the timing of the saccade because it mainly depends on the perceived velocity between 100 and 200 ms before the tap. We found no similar modulation of the timing of the corrective saccade on the velocity judgments, suggesting that here the information is integrated uniformly across the whole trial.

## Discussion

We confirmed that making corrective saccades while tracking a moving target affects judgments about such targets’ velocities^16^. The occurrence of forward saccades made the targets appear to move faster than identical targets tracked with pure pursuit. Backward saccades had the opposite effect (Fig. 3). The change in perceived velocity was reflected in performance when participants had to intercept the moving targets. Targets tracked with additional forward saccades were hit further ahead than ones tracked with pure pursuit. Backward saccades led to tapping at positions further behind the target than is observed with pure pursuit (Fig. 4). The magnitude of changes in perceived velocity were not correlated with the magnitude of changes in the interception behavior (across participants; Fig. 6a). This may be because the magnitude of changes in interception depended on the timing of the saccades (Fig. 6b). The timing of corrective saccades (Fig. 5a) and that of interception (Fig. 5c) were adjusted to maximize the time without corrective saccades just before the moment of the interception.

### Corrective saccades affecting a general velocity signal

The existence of a velocity representation that is independent of the instantaneous oculomotor behavior has been proposed to explain the perceptual effect that we replicated in the current paper^16^. That work modeled the effect of corrective saccades by a dynamic integration of those two signals, which is distorted around the time of corrective saccades, because the retinal signal becomes less reliable due to active and passive saccadic suppression processes^29–31^. The existence of such a general velocity signal is a rather new idea, as classically saccadic and pursuit eye movements were mainly studied in isolation and thought to belong to different subsystems (see^9^ for an overview) that influence velocity judgments in completely different ways. However, a recent study has shown that both saccades and pursuit seem to be affected by a shared velocity signal^32^. Our current experiment provides further evidence for such a theory. We were able to replicate the perceptual effect of corrective saccades and could extend this finding by observing a similar effect in the interception task. This indicates that corrective saccades might have an impact on a more general velocity estimate of the target that is not only used for velocity judgments, but also for the prediction of the interception point when tapping with the hand and probably also for other actions.

Changes in the velocity estimates are also reflected in the hand’s velocity shortly before the tap^27^. We found that trials with additional forward saccades had a higher hand velocity shortly before the tap, whereas trials with additional backward saccades had a lower one. One could argue that this might be due to the effect of corrective saccades on the time it took to intercept the target. Targets in trials with corrective saccades were intercepted later, and therefore at more eccentric positions that could be related with a higher velocity of the hand. However, targets were intercepted later and therefore at more eccentric positions for trials with both forward and backward saccades, whereas the velocity just before intercepting the targets was affected in different directions for the two kinds of saccades. Thus, the adjustment of the hand velocity seems to be a consequence of the change in the perceived velocity of the target.

While we observed similar effects of the corrective saccade in the interception task as in the judgment task, the timing of the corrective saccades seems to modulate the effect on interception. Interception behavior seems to be guided by a constant updating of judged velocity and position of the target until about 100 ms before the tap^19^. Therefore, the tapping error is most sensitive to saccades that occur at that time (Fig. 6b). Later information cannot influence the tap, and earlier information leads to errors that can be corrected before the tap. If a corrective saccade occurs early enough, updating the interception endpoint with the information provided after the corrective saccade leads to a comparable performance to that in the pure pursuit trials. In contrast, for perceptual judgments the information about target velocity is probably accumulated across the whole trial to obtain a precise judgment. This integration of information across the whole trial can explain why saccades at different points in time have a similar effect on the final velocity judgment (Fig. 6c). It is also consistent with the finding that saccades of similar sizes have less effect on the perceived velocity when the trials are longer^16^. Thus, corrective saccades influence the representation of target velocity. How the information is used depends on the task. While the interception behavior is strongly influenced by a change in this velocity representation around 100 to 200 ms before the tap, for perceptual judgments the information is integrated uniformly across the whole trial.

### Relation between pursuit quality and performance in interception

Despite the potential costs of drastically changing the retinal input by one’s own eye movements, directing gaze at an object that we are aiming for is beneficial for the accuracy of arm movements^33,34^. Our current findings suggest that the potential costs are avoided by directing one’s gaze early during the aiming movement. An additional benefit of directing one’s gaze at the object of interest is that if the object is moving, tracking the moving object will improve our ability to predict its trajectory^5^ and to intercept the object at the correct place and time^3,6^.

**Figure 7:**
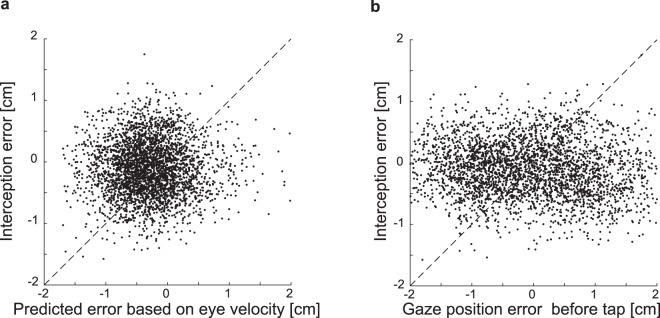
Relation between oculomotor behavior and interception performance. **(a)** Errors in the interception task as a function of the expected endpoints based on the eye velocity 100 ms before the tap. Dots represent all individual pure pursuit trials of all participants. The dashed black line is the unity line. (**b**) Errors in the interception task as a function of the gaze position error (with respect to the target) 100 ms before the tap. The dotted line is the unity line. Positive values indicate errors ahead of the target, negative values indicate errors behind the target.

Whether the quality of tracking is related to performance in interception tasks is still under debate. Some studies have shown a relationship between strategic eye movement behavior and task performance^6,35,36^, whereas others report a benefit of tracking but no correlation between tracking accuracy and task performance^8,37^. To test whether we could use the eye velocity on individual trials to predict the interception error on those trials, we estimated the gaze velocity 100 ms before the tap by taking the mean of the velocity between 120 and 80 ms before the tap, subtracting the target velocity from this, and multiplying the outcome (a measure of pursuit error) by 0.1 s. Our results show that neither this error measure based of eye velocity nor the (signed) gaze position error at the crucial moment, 100 ms before the tap, is predictive of the interception error (Fig. 7). Therefore, our results suggest that on a trial-by-trial basis accurate eye movements are not necessary to perform accurate interceptive movements. Variability in eye velocity can be compensated for by combining the eye velocity signals with retinal information^12^. The measurement of instantaneous eye velocity is therefore dissociable from the representation of the target movement that is used for interception. The correlation that has previously been reported^6,35,36^ could be explained by the fact that overall performance of pursuit and interception behavior might both depend on how well one can predict future target movements. Predictions are necessary for both oculomotor^38–40^ and interception control^41^ so a correlation between those broad measures might not come as a surprise. At the single trial level motor noise and different compensation mechanisms, like the adjustment of pursuit velocity based on target position^42,43^, disrupt the direct relation between the current eye velocity and the target velocity representation used to predict the interception endpoint.

### Timing adjustments of corrective saccades and interceptions

In step-ramp paradigms forward saccades typically happen later than backward saccades^44^. These differences in latency are related to the movement of the stimulus. In the case of short target crossing times, the target is passing through the fovea around the time of pursuit initiation. Thus, error signals at this point are not large, so the decision to trigger a corrective saccade is delayed until error signals accumulate by the target moving further away^26^. For long target crossing times, the eye is initially well ahead of the target and pursuing the target prevents its motion from quickly decreasing the position error. Thus, earlier saccades prevent long episodes with large position errors in this case. However, target eccentricity is not the only factor that influences saccadic latencies because both pursuit onset and saccadic latencies were shorter in the interception task (Fig. 5a). This general reduction of oculomotor latencies was probably due to the time pressure in the interception task. Participants received feedback about their success and were therefore highly engaged to be as fast and accurate as possible. In the judgment task they just had to follow the target with the eyes, with no time pressure to give their judgment and no explicit feedback about their pursuit.

There were some specific adjustments in the oculomotor and interception behavior that point to a general strategy of trying to maximize the time without a corrective saccade before the tap. The shorter saccadic latencies could to some extent be explained by a general speeding of oculomotor latencies, including an earlier start of the pursuit response. However, when we analyzed the eye crossing times, which are considered to be related to the decision to make catch-up saccades during pursuit^24^, we found a difference between forward and backward saccades. There was no change in eye crossing time for backward saccades, suggesting that the latency change of backward saccades was probably related to the general decrease of oculomotor latencies. In contrast, for trials with forward saccades, which typically happen later in the trial, there were some strategic adjustments. First, forward saccades occurred with significantly smaller eye crossing times in the interception task (Fig. 5b). This indicates that they were triggered when there was a smaller potential error signal, so that they occur earlier during the trial. Second, participants took more time until they tapped in trials with forward saccades (Fig. 5c). Thus, in line with the results of previous studies^45^, participants seem to adjust their oculomotor behavior to the task they need to deal with. Minimizing the occurrence of corrective saccades before interception is a useful strategy to maximize spatial resolution at the time close before the tap^4^ to gain reliable information at this crucial moment^28^. It also avoids the effects of saccadic suppression or mislocalization^30,31^, which starts already roughly 100 ms before the execution of the saccade^31,46^.

In summary, besides replicating the effects of corrective saccades on velocity judgments we could demonstrate that interception performance is affected in a similar manner. However, the effects of corrective saccades on interception errors were not correlated with their effects on velocity judgments. We attribute this to information about velocity being used differently for the two tasks: integrated across the whole trial for the velocity judgments but estimated at each moment for interception. Despite the identical target movements in the interception and judgment tasks, the corrective saccades occurred earlier in interception, probably to maximize the time without a corrective saccade just before interception.

## Data Availability

Data supporting these findings are available at zenodo.org (10.5281/zenodo.2566255).
